# Severe Lenalidomide-Associated Hyperbilirubinemia

**DOI:** 10.7759/cureus.34408

**Published:** 2023-01-30

**Authors:** Daniel T Gildea, Joseph L Roswarski

**Affiliations:** 1 Internal Medicine, MedStar Georgetown University Hospital, Washington, D.C., USA; 2 Hematology and Oncology, MedStar Georgetown University Hospital, Washington, D.C., USA

**Keywords:** oncology, treatment related toxicity, cholestatic liver injury, extra-medullary plasmacytoma, immunomodulatory imide drugs, direct hyperbilirubinemia, hepatology

## Abstract

Immunomodulatory drugs (IMids), such as thalidomide and lenalidomide, are used to treat plasma cell neoplasms and B-cell malignancies. We present a case of severe direct hyperbilirubinemia in a patient taking lenalidomide-based therapy for plasmacytoma. Imaging was unrevealing, and liver biopsy showed only mild sinusoidal dilation. Roussel Uclaf Causality Assessment (RUCAM) score was 6, indicating lenalidomide was a probable cause of the injury. To our knowledge, this is the highest reported direct bilirubin regarding lenalidomide drug-induced liver injury (DILI), with a peak bilirubin of 41mg/dL. While a clear pathophysiology was not identified, this case provides important considerations regarding lenalidomide safety.

## Introduction

Lenalidomide is an immunomodulatory drug that has been increasingly used in plasma cell neoplasms, B-cell malignancies, and myelodysplastic syndrome. It is thought to have a multifaceted method of action, including inhibiting inflammatory cytokines such as TNF-alpha and IL-6, T-cell stimulation, augmenting natural killer cell function, inhibition of angiogenesis, and direct anti-tumor activity [1]. The method of action involves activation of cereblon to induce proteasomal destruction of B-cell transcription factors [2] and inhibition of NF-kappa B [3]. There have been reports of lenalidomide causing cholestatic liver injury [4] and mild bilirubin elevations, but extreme hyperbilirubinemia as seen in this case has yet to be reported.

## Case presentation

At our institution, we admitted a 46-year-old black male with a past medical history of high-grade anaplastic plasmacytoma, developmental delay, and hypertension for acute kidney injury (AKI) and abnormal liver-associated enzymes. He had been recently started on therapy for his anaplastic plasmacytoma and was admitted on cycle 2, day 17 of treatment with carfilzomib, lenalidomide, cyclophosphamide, and dexamethasone.

Prior to this admission, he received one cycle of carfilzomib, cyclophosphamide, and dexamethasone. He tolerated this well and pre-cycle-2 labs, including blood counts and liver/kidney function, were normal. To augment response to chemotherapy, lenalidomide 25mg orally given from days one through 21 of the cycle was added to the regimen for cycle 2. He presented to our hospital on cycle 2, day 17 for abnormal labs (Table 1). Upon admission (day one), creatinine, blood urea nitrogen (BUN), total bilirubin, and direct bilirubin were all elevated. Alanine aminotransferase (ALT) and alkaline phosphatase were slightly increased from their previous baseline, as well. On exam, the patient appeared somnolent and jaundiced, but there were no other focal findings. His mother noted that he had been experiencing nausea and vomiting with poor oral intake for two weeks prior to admission; given this, his AKI was thought to be pre-renal secondary to dehydration. Lenalidomide was held on admission. Right upper quadrant ultrasound noted a normal liver exam without ductal dilation or abnormal flow. A non-contrast CT scan of the chest/abdomen/pelvis was also unrevealing. Labs were trended daily (Table 1). There was a daily increase in his direct bilirubin level until it reached a peak above the upper detectable limit of our institution (>22.5mg/dL) from hospital days four to six, with subsequent slow resolution. The patient’s creatinine level stayed elevated from hospital day one to 10, when it began downtrending. The aspartate aminotransferase (AST), ALT, and alkaline phosphatase did not have significant elevations.

**Table 1:** Timing of Liver-Associated Enzyme Elevations During Hospitalization Ref.: Normal reference range Pre-C2 labs: Labwork completed prior to cycle 2 (C2) of lenalidomide dose D: Denotes day of hospitalization, with D1 denoting hospital day 1 BUN: Blood urea nitrogen, AST: Aspartate aminotransferase, ALT: Alanine aminotransferase

	Ref.	Pre-C2 labs	D1	D2	D3	D4	D5	D6	D7	D8	D9	D10	D11	D12	D13
Total Bilirubin (mg/dL)	0.1 – 1.2	0.6	5.4	5.0	7.4	33.1	41.9	34.4	24.9	7.0	4.0	3.5	2.7	2.4	2.1
Direct Bilirubin (mg/dL)	<0.3		4.7	4.5	6.2	>22.5	>22.5	>22.5	18.40	5.9	3.3	2.9	2.3		
Creatinine (mg/dL)	0.74 – 1.35	0.82	3.34	3.65	3.59	3.42	3.27	3.63	3.75	3.65	3.23	3.03		2.73	2.42
BUN (mg/dL)	6 - 24	16	67	76	70	87	96	101	85	94	77	59		45	34
AST (units/L)	8 - 33	14	25	44	41	18	34	29	23	15	24	36	32	32	16
ALT (units/L)	4 - 36	11	57	59	62	44	44	42	39	29	30	41	41	38	26
Alkaline Phosphatase (units/L)	44 - 147	69	130	130	142	154	150	143	159	130	124	128	112	110	93

A magnetic resonance cholangiopancreatography (MRCP) noted no ductal dilation and no intrahepatic or extrahepatic masses. Liver biopsy was performed during the resolution phase of hyperbilirubinemia on hospital day 10, showing unremarkable liver parenchyma with no steatosis, hepatitis, cholestasis or necrosis. Mild sinusoidal dilatation without congestion was noted. Viral studies, including hepatitis A, B, and C, Epstein-Barr virus (EBV), herpes simplex virus (HSV), and cytomegalovirus (CMV) were all negative. The patient became noticeably more fatigued and confused during his hospital stay, with resolution to his baseline prior to discharge, mirroring lab improvement. Given the improvement with fluids and the holding of lenalidomide, the patient was discharged home with oncology follow-up to discuss potential rechallenge with a lower lenalidomide dose versus discontinuing lenalidomide entirely from the regimen.

## Discussion

Anaplastic plasmacytoma is a rare variant of extramedullary plasma cell neoplasms. In our case, the patient was treated with a multiagent regimen including an immunomodulatory drug (IMid), lenalidomide, and proteasome inhibitor, similar to what is done for multiple myeloma. The patient presented with AKI and progressive direct hyperbilirubinemia. He did not have hyperbilirubinemia in the previous cycle when lenalidomide was absent. The Roussel Uclaf Causality Assessment Method (RUCAM), an objective measure of likelihood of drug-induced liver injury (DILI), was used to calculate likelihood of DILI in this case. This score takes into account various factors, including time of onset, known drug hepatotoxicity, other possible etiologies, and more [5]. In general, a score of 9 or more indicates high likelihood, while 6-8 means probable, 3-5 is possible, and 1-2 is unlikely, with DILI being excluded at any lower score [5]. In this case RUCAM was calculated at 6, indicating that DILI secondary to lenalidomide is probable. While there have been a handful of other reports of lenalidomide causing cholestatic liver injury [4], direct hyperbilirubinemia [6], unmasking Gilbert’s with indirect hyperbilirubinemia [7,8], idiosyncratic predominately hepatocellular injury [9], and rarely acute liver failure [4], to our knowledge this level of extremely elevated direct hyperbilirubinemia due to lenalidomide has not been reported in the literature. Notably, the patient had developed a significant AKI soon after starting lenalidomide, and as the drug is mainly renally excreted [6], the patient may have experienced a significant physiologic overdose, leading to more profound hyperbilirubinemia compared to previous reports. There have been reports of lenalidomide-induced acute renal failure [10], but dehydration and pre-renal etiology were suspected in our case. Additionally, AKI-induced lenalidomide hepatotoxicity has been suggested in a prior case report [11].

The pathophysiologic effect of lenalidomide on the liver is unclear. The liver imaging by two different modalities argued against mechanical obstruction, and viral serologies were negative. The liver biopsy, albeit performed during both laboratory and clinical improvement, noted only mild sinusoidal dilation without congestion. This could suggest that the mechanism of injury was sinusoidal obstruction syndrome, which appeared to resolve rapidly with lenalidomide discontinuation. There have been reported cases of lenalidomide-induced intrahepatic cholestasis with direct hyperbilirubinemia, with bilirubin levels that follow a similar laboratory trend to our patient’s levels. However, these cases did not cause extreme hyperbilirubinemia and had significant elevations of alkaline phosphatase [12], which was not seen in our patient.

## Conclusions

Immunomodulatory drugs such as thalidomide, lenalidomide, and pomalidomide are frequently used for various hematologic malignancies, particularly plasma cell neoplasms. Compared with conventional cytotoxic chemotherapy, they have a distinct toxicity profile for which the treating physician should exercise caution. Previously documented adverse hepatic effects include mild cholestatic abnormalities. We are adding to the literature of what is known about lenalidomide toxicity and posit that the development of AKI is a mechanism for more profound hepatic toxicity to occur.
